# The management of esophago-gastric necrosis due to caustics ingestion: Anastomotic reinforcement with Cyanoacrylate glue and damage control with Vacuum Assisted Closure Therapy—A case report

**DOI:** 10.1016/j.ijscr.2019.06.032

**Published:** 2019-06-28

**Authors:** A. Picciariello, V. Papagni, G. Martines, N. Palasciano, D.F. Altomare

**Affiliations:** Department of Emergency and Organ Transplantation, University Aldo Moro of Bari, Bari, Italy

**Keywords:** Gastric necrosis, Cyanoacrylate glue, Esophago-jejunum anastomosis, Damage control, Vacuum assisted closure

## Abstract

**Introduction:**

The surgical treatment of a complete gastric necrosis due to caustic ingestion is extremely challenging and life threatening. In this emergency scenario, a first-time reconstruction of the gastrointestinal tract is often dangerous for the patient because of the high risk of infections and anastomosis leakage. Literature lacks of clear indications for the management of this condition.

**Presentation of case:**

Male patient with history of major depression disorder was admitted to our Emergency Unit after the ingestion of muriatic acid. CT scan showed massive pneumo-peritoneum with esophago-gastric thickening. Free fluids in the abdominal cavity were detected. Intraoperative finding was a complete necrosis of the stomach and corrosion of the lower esophagus.

**Discussion:**

In this case report we proposed a first approach with the drainage and lavage of the abdomen cavity. Then, an esophago-jejunum anastomosis reinforced by Cyanoacrylate glue was performed and a damage control with VAC therapy (Vacuum Assisted Closure) was carried out.

**Conclusion:**

Cyanoacrylate glue could be considered useful and efficient in the reinforcement of anastomosis even in emergency surgical procedures. Damage control using VAC allows to keep a good control of the surgery performed.

## Highlights

• Optimal timing to treat esophago-gastric necrosis due to caustic ingestion.
• Proposal of three steps approach to manage esophago-gastric necrosis.
• Use of Cyanoacrylate glue to reinforce anastomosis.
• Vacuum Assisted Closure for damage control after surgery.

## 1 Introduction

Upper gastrointestinal injuries due to caustic ingestion are rare surgical emergencies often associated to intentional suicide attempts in adults [1,2]. These emergencies usually concern patients with an age starting from 21 years old [3].

The scenario after a caustic ingestion is extremely various, ranging from a single perforation of the stomach or an esophageal stricture to a complete gastric necrosis [4].

Patients survival after severe caustic gastrointestinal injuries depends on the time frame between ingestion and surgical treatment [5].

Esophagogastroduodenoscopy represents the **gold** standard to evaluate the severity and the extension of the injury and it also allows to choose the best treatment for patients [6].

It is quite debated in literature what is the best surgical procedure to treat this condition; in fact both one stage and two stages procedures have been proposed [6,7].

We aimed to report our experience in the management of a patient with severe full-thickness gastric wall necrosis associated with partial low esophageal injury after the ingestion of hydrochloric acid.

A two-time surgery was performed using cyanoacrylate glue for the reinforcement of the esophaho-jejunal anastomosis. Furthermore, we carried out a damage control using VAC (Vacuum Assisted Closure) therapy in order to keep a good control of surgery.

This case report is written according to SCARE criteria [8].

## 2 Presentation of case

A 64 years old male was admitted to our Emergency Unit showing anxiety, confusion and agitation, pharyngeal burns and epigastric pain. GCS 15.

The anamnesis was collected by his relatives who reported muriatic acid ingestion about 5 h before First Aid admission. Furthermore, the patient has been in a state of major depression for 10 years. No other comorbidities or previous surgeries were reported.

Patient vital signs were BP 100/55 mmHg, HR 125/min, RR 22/min, temperature 37 °C, weight 95 Kg, height 180 cm.

At physical examination: tenderness of the abdomen, positive Blumberg sign, absence of bowel sounds.

The hemogasanalysis showed a metabolic acidosis (pH 7.14) with a slight hypercalcaemia (1,29 mmol/L) and a hyperchloremia (118 mmol/L).

Blood tests showed neutrophilic leukocytosis and an increase of C-reactive protein (112 mg/L), Hb 16.7 g/dl and Hct 51%, lactic acid 1.8 mmol/L.

At First Aid an ECG was performed (sinus tachycardia, 117 bpm) and the patient underwent a chest x-ray and an Esophagogastroduodenoscopy (EGDS) showing an extensive mucosal necrosis (Zargar 3B).

The abdomen CT scan demonstrated a massive pneumo-peritoneum with esophago-gastric thickening. Free fluids in the abdominal cavity around the stomach were detected.

**Fig. 1:**
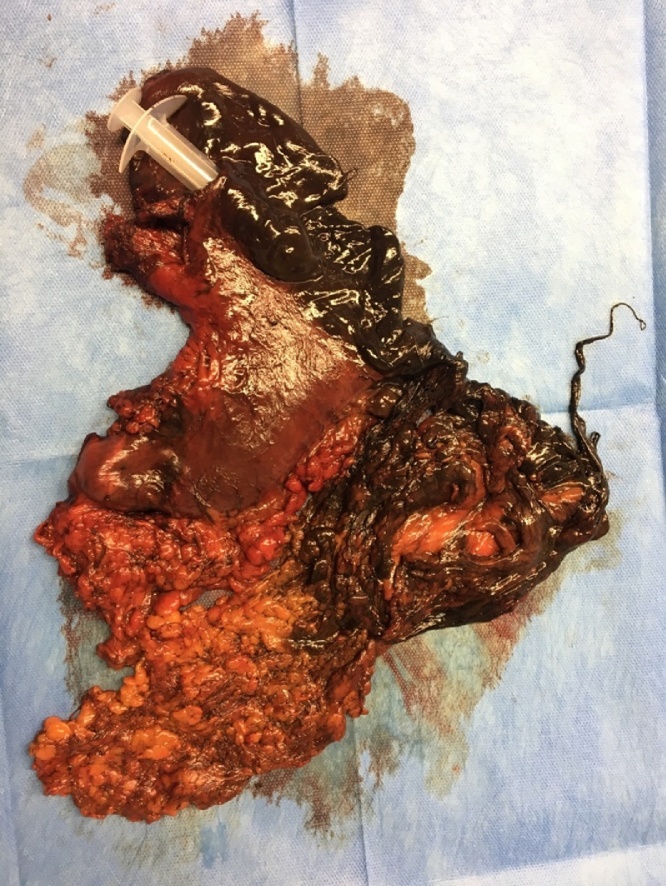
Specimen Total gastrectomy with omentectomy.

The patient underwent emergency surgery (ASA IV E) and during the first operation the abdomen was explored with evidence of a large aperture of the posterior-lateral gastric wall and total gastric necrosis with the presence of ingested material. Considering the patients’ poor performance status, as first approach three tubular drains were put in the abdomen after a lavage; the aperture on the posterior wall of the stomach was closed and the necrotic tissues was removed. In the second operation (18 h after) a total gastrectomy with omentectomy (Fig. 1) and a stapled functional side-to side esophago-jejunal (E-J) anastomosis on the anterior esophageal wall were performed. An end to side jejuno-jejunal anastomosis was carried out to restore the gastrointestinal transit.

**Fig. 2:**
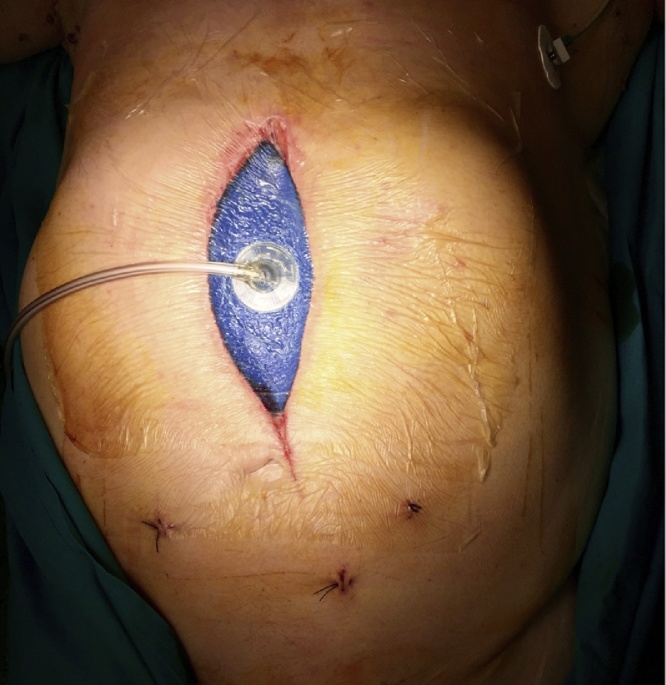
Vacuum Assisted Closure Therapy.

Cyanoacrilate glue was used to reinforce the E-J anastomosis and damage control with VAC (Vacuum Assisted Closure) (Fig. 2) was carried out in order to keep a good control and to allow a second look surgery.

After 48 h VAC System was removed and the anastomosis was checked with an esophagojejunoscopy with an air leak test (negative). The abdomen was closed with interrupted Vicryl 1 for the fascia.

On postoperative day (POD) 5 the patient underwent parenteral nutrition in the Intensive Care Unit and on POD 8 a temporary tracheostomy was performed and the patient started to drink.

On POD 14 he had a pneumonia treated by antibiotic therapy and on 22 POD he was moved to the Psychiatry Unit where he started a therapy for the major depression and a semisolid diet.

**Fig. 3:**
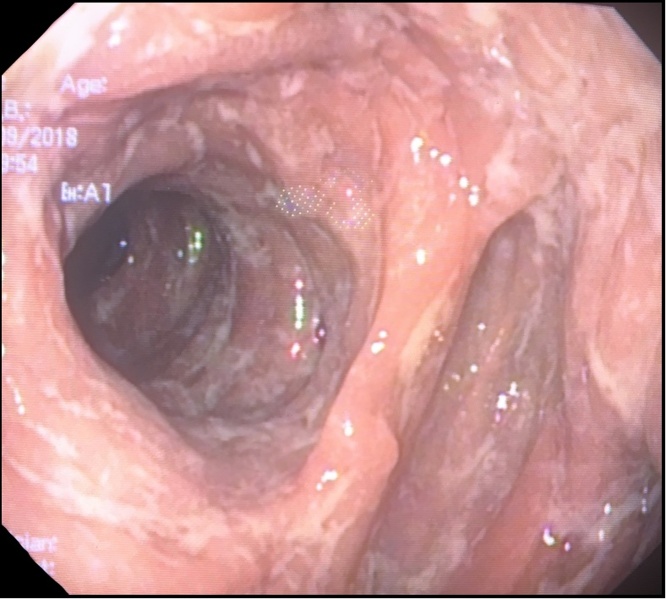
Endoscopic control on POD 26.

On POD 26 a further esophagojejunoscopy demonstrated the absence of leakage and a good transit through the anastomosis (Fig. 3). The wound healing was completely normal and the patient was discharged on POD 33.

## 3 Discussion

Massive Ingestion of corrosive substances for suicidal intent in adults represents a surgical emergency with a high rate of mortality (about 20%) and long-term consequences [9]. The real prevalence of these injuries is underrated because literature lacks of reviews and includes only random papers and case reports/case series.

Injuries from caustic substances are more common in Western countries than in developing countries where people can easily find acids which are used for suicidal intent [10].

Lesions after caustic ingestion are more sever in adults, maybe due to the massive amount of caustic ingested [1]. Among devastating consequences due to caustic ingestion, severe injuries, such as necrosis of the oesophagus and/or the stomach, can occur leading to short and long term life-threatening complications [11].

Most of times, the first approach is a life-saving medical therapy performed to keep the patient stable (e.v fluids, antibiotics and electrolytes correction) and, if necessary, an orotracheal intubation to assess airway safety [9].

**Fig. 4:**
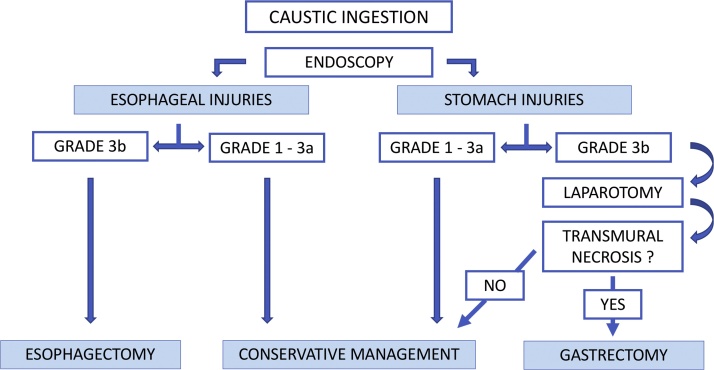
Algorithm for emergency management of caustic injuries.

Even if there are no strict guidelines regarding the indication of endoscopy after the ingestion of a large amount of corrosives, an EGDS, in absence of a third degree burn of the hypopharynx, is useful to stage the injury and to make the best choice to treat it [6]. Grade 3B injury, according to Zargar endoscopic classification (Table1), is a life-threatening condition and an immediate laparotomy is mandatory [11] (Fig. 4).

**Table 1:** Zargar's grading classification of mucosal injury caused by ingestion of caustic substances.

GRADE	DESCRIPTION
0	Normal examination
1	Edema and hyperemia of the mucosa
2a	Superficial ulceration, erosions, friability, blisters, exudates, hemorrhages, whitish membranes
2b	Grade 2° + deep discrete of circumferential ulceration
3a	Small scattered areas of multiple ulceration and areas of necrosis with brown/black or greyish discoloration
3b	Extensive necrosis
4	Perforation

According to Cattan et al. the surgical treatment of severe caustic injuries should performed as soon as possible in order to improve the prognosis of patients [12].

Data from literature show the lack of a standardized surgical procedure for necrosis of stomach and/or oesophagus due to caustics ingestion. Some authors report good short and long term outcomes of one-time surgical treatment with esophagojejunal reconstruction after total gastrectomy [11,13].

On the other hand, damage control surgery (DCS) [14] with the use of VAC system could be a valid approach for life-threatening conditions. In fact, DCS involves more steps for the treatment and, usually, the main surgical procedure is performed only when patients are stable. In this way, the rate of adverse complications after surgery could be lower.

In this case we report our experience using a three steps approach for complete stomach necrosis due to acid ingestion. Firstly, a drainage of the abdomen cavity was performed cleaning the abdomen by the necrotic tissue and closing the aperture on the posterior wall of the stomach with a running suture. During the second operation a total gastrectomy and esophago-jejunum anastomosis reinforced by Cyanoacrylate glue was carried out. Cyanoacrylate glue is a synthetic glue with sealing, adhesive and hemostatic properties widely used in elective surgery [15]. After the operation, the patient underwent damage control with VAC therapy that allowed us to check the condition of the anastomosis after 48 h.

VAC could be considered very helpful for critical patients since it allows a faster abdominal closure and an earlier discharge from the Intensive care Unit [16,17].

## 4 Conclusion

Ingestion of caustic substances has devastating consequences on the esophagus and the stomach and often emergency surgery is required. Cyanoacrylate glue could be considered useful and efficient in the reinforcement of anastomosis even in emergency surgical procedures. Damage control using VAC allows to have a good control of the surgery performed and to make a revision of the abdomen two days after the critical surgical procedure and before the closure of the abdomen.

## Conflicts of interest

No conflict of interest to declaire.

## Funding

No founding received.

## Ethical approval

Ethical approval was obtained by International Review Board of Azienda Ospedaliera Univeristaria –Policlinico di Bari, P.zza G. Cesare, Bari, Italy

## Consent

Written informed consent was obtained from the patient for publication of this case report and accompanying images. A copy of the written consent is available for review by the Editor-in-Chief of this journal on request.

## Author contribution

Arcangelo Picciariello: conception of study design, data collection, analysis, manuscript writing, revision and manuscript submission.

Papagni Vincenzo: conception of study design, data collection, analysis, revision of the manuscript.

Gennaro Martines: critical revision of the manuscript, approved the final version of the manuscript for submission.

Nicola Palasciano: data collection, analysis, manuscript writing and revision.

Donato F. Altomare: manuscript writing, drafting, revising of the manuscript and participation in the care of the patient.

## Registration of research studies

This study does not require the registration.

## Guarantor

Arcangelo Picciariello, MD.

## Provenance and peer review

Not commissioned, externally peer-reviewed.
